# Scarlet Fever Outbreaks

**Published:** 1919-11-22

**Authors:** 


### SCARLET FEVER OUTBREAKS.

### With Special Reference to the Latest Epidemics.

THE two recent outbreaks of scarlet fever at Belfast and Birmingham are of interest to those conversant with the epidemiology of this disease, as they so exactly fall in with the type almost invariably found in epidemics, and occur at the time of year at which the disease is ever at its maximum. Also, in both cases a sudden wave of scarlet fever is occurring after a more or less prolonged depression. A divergence occurs only in the fact that the climatic conditions are not quite in accordance with those which are usually regarded as most favourable.

As to the type of disease in these two epidemics; in both cases it is mild. The patients not being in any way ill continue their attendance at school, and thereby infect their fellow scholars, while those whose illness is sufficient to need medical attention are less likely to be diagnosed. Such mild epidemics may, however, on occasions develop into the severe type, the change usually marking the termination of the particular outbreak, for the patients now being extremely ill, are automatically kept from contact with other children, and also leave little doubt as to diagnosis.

### THE TIME OF YEAR.

That the outbreak should be at its maximum in October is also quite in accordance with precedent. The curves for the mortality of scarlet fever in England show a minimum in the spring, March, and April being the actual months, from which time there is a steady rise till October, and after that a fall till March again. The curves for incidence of the disease follow the same tendency but often show considerable variations, so that it comes about that the case mortality may be lower in October than in March, either because the disease in the spring is more virulent than in the Autumn, or perhaps because the vitality of the children is lower in the spring. That increased virulence should go with a reduced number of cases is quite in accord with the general epidemiology. The actual figures of new cases at Birmingham are:—Week ending October 4, 82; week ending October 11, 134; week ending October 18, 144. The number of patients in hospital on October 18 was 409 as compared with 107, the figure for the corresponding week last year.

In both cases the period since the previous epidemic is long—five years in the case of Birmingham—and at Belfast there is no record of a previous outbreak on so large a scale. Three years is the critical age with regard to scarlet fever, once that age is passed children become more and more immune both to contracting the disease and also to the disease itself when contracted. From this it may be argued that from three to six years after one epidemic there will be the maximum of susceptible persons, for a high degree of immunity is undoubtedly conferred by an attack.

As with most of these diseases which owe their dissemination to breath-to-breath infection, and in which, though milk, dirty clothes, etc., play an undoubted *rôle*, there is little doubt that direct infection is the principal factor, scarlatina tends to be most prevalent at these seasons when people congregate indoors for warmth and shelter. The crowding is an obvious factor, and in the moist atmosphere resulting from the crowd and the state of the weather, the causal organism is enabled to retain its vitality for a longer period than when it stands a chance of dessication.

The bacteriology of the disease is most unsatisfactory, as is not unnatural when it is considered that the throat is on all sides looked upon as the primary focus. But one may observe in passing that it is quite the exception to obtain a culture from the throat either in health or disease in which one type and one only of the streptococcus is found; hence, only in the hands of a patient enthusiast is a differential diagnosis of pathogenic from non-pathogenic organism possible.

### THE CHIEF DIFFICULTY.

The chief difficulty in dealing with outbreaks of scarlet fever, or, in fact, with outbreaks of any of the exanthemata, is accommodation. It is this factor which calls attention to the outbreak at Belfast, where the Corporation Fever Hospital has a nominal 268 beds, while the number of cases of scarlatina alone is 581. In this case the overflow is being dealt with by the Guardians, who have been receiving cases into the Poor-law infirmary. That a sufficient accommodation should be available is an obvious necessity, but it is hardly a matter for wonder that when the total number of cases needing treatment suddenly rises to double the number of beds previously found to be sufficient, a state of some disorganisation should result. In the Army we often met this sort of case, and by the erecting of spare tents, or by the taking over of tents from other people, managed to keep things going. The staff, of course, just had to work overtime. But in a civilised community this or similar proceedings are not possible; one cannot take over a big house or hotel, nor can one fill the public parks with hospital marquees. At the same time, to keep large surplus buildings ready and staffed is a matter which would cause the rate-payer many searchings of heart, though this is the line of action most usually adopted. But nowadays, when the transport of sick persons has reached so high a state of perfection, it should surely be possible to arrange for excess patients from one district to be moved to and taken care of by the institutions of some other area. This should the more easily be done as epidemics of the acute exanthemata are seldom pandemics, but are restricted to one separate district, and the idea of such case distribution is worthy of further consideration.

